# Revolutionizing Neonatal Care: A Comprehensive Review of Intact Cord Resuscitation in Newborns

**DOI:** 10.7759/cureus.68924

**Published:** 2024-09-08

**Authors:** Sai Bhavani Manchineni, Revat J Meshram

**Affiliations:** 1 Pediatrics, Jawaharlal Nehru Medical College, Datta Meghe Institute of Higher Education and Research, Wardha, IND

**Keywords:** delayed cord clamping, intact cord resuscitation, neonatal outcomes, neonatal resuscitation techniques, physiological transition, umbilical cord management

## Abstract

Neonatal resuscitation is a critical procedure aimed at ensuring the successful transition of newborns from intrauterine to extrauterine life. Traditionally, this involves immediate clamping and cutting of the umbilical cord, but recent advances have introduced intact cord resuscitation (ICR) as an alternative approach. This review aims to comprehensively analyze ICR, exploring its evolution, scientific basis, and clinical evidence. It seeks to evaluate the benefits and challenges associated with ICR and assess its impact on neonatal outcomes compared to traditional practices. A thorough review of the literature was conducted, including historical perspectives on neonatal resuscitation, the physiological rationale behind ICR, and critical clinical studies and trials. Current guidelines and recommendations were also examined, along with technological advancements and practical implementation issues. Evidence indicates that ICR offers significant benefits, including improved blood volume, better cardiovascular stability, and reduced risk of anemia in newborns. Comparative studies suggest that ICR can enhance neonatal outcomes and support a smoother transition to extrauterine life. Despite these benefits, challenges related to implementation and adherence to new practices persist. ICR represents a promising advancement in neonatal care, potentially improving newborns' health outcomes. Continued research and refinement of guidelines are necessary to fully integrate ICR into standard practice and address existing implementation challenges. This review highlights the need for ongoing evaluation and adaptation of resuscitation practices to optimize neonatal health and care.

## Introduction and background

Neonatal resuscitation is a cornerstone of obstetric and pediatric care, crucial for ensuring that newborns successfully transition from intrauterine to extrauterine life [1]. Traditionally, this process has been governed by a set of standardized protocols that include immediate clamping and cutting of the umbilical cord, followed by the rapid assessment and stabilization of the infant. This conventional approach has been predicated on the belief that early intervention was necessary to minimize risks and facilitate the newborn’s adaptation to life outside the womb [2]. Historically, the focus has been on swift interventions to prevent birth complications and promote immediate respiratory and circulatory stability [3]. In recent years, however, neonatal resuscitation has undergone significant scrutiny and revision, particularly with the advent of intact cord resuscitation (ICR). ICR, often called delayed cord clamping, involves postponing the clamping and cutting of the umbilical cord until after initial resuscitation efforts are completed [4]. This approach aims to take full advantage of the placental blood transfer, which can enhance neonatal outcomes by improving blood volume and reducing the risk of anemia. The evolution of ICR reflects a paradigm shift driven by emerging evidence suggesting that this method may offer substantial benefits over traditional practices [5].

The importance of reviewing current practices and evidence related to ICR is underscored by the ongoing advancements in neonatal care and the need to optimize resuscitation strategies [5]. As our understanding of neonatal physiology and resuscitation techniques continues to evolve, it becomes essential to critically evaluate the impact of ICR on clinical outcomes. This includes assessing its effectiveness in improving newborn health, understanding its implications for current resuscitation protocols, and identifying potential areas for further research [5]. The purpose of this review is to provide a thorough examination of ICR in newborns. By exploring the historical context and evolution of ICR, we aim to contextualize its development within the broader framework of neonatal care. This review will delve into the scientific basis of ICR, assess the clinical evidence supporting its benefits, and evaluate current guidelines and recommendations. Through a comprehensive analysis of recent advancements and identification of gaps in knowledge, this review seeks to contribute to refining neonatal resuscitation practices and enhancing the quality of care provided to newborns.

## Review

Historical perspective

Traditional Neonatal Resuscitation Practices

Neonatal resuscitation is a critical intervention for newborns who are unable to initiate effective breathing at birth. Standard practices are guided by protocols established by organizations such as the American Heart Association (AHA) and the American Academy of Pediatrics (AAP). The primary goals are to restore adequate breathing, heart rate, and oxygenation while preventing hypoxic-ischemic injury to vital organs [6]. Critical steps in standard neonatal resuscitation include an initial assessment followed by the initial steps: providing warmth, positioning the infant, clearing the airway if necessary, drying the infant, and stimulating breathing. Positive-pressure ventilation is initiated if the infant does not begin breathing adequately, typically using a bag-mask device. Chest compressions are performed if the heart rate remains below 60 beats per minute despite adequate ventilation. In cases of severe asphyxia or shock, medications such as epinephrine and volume expanders may be necessary. Continuous monitoring and support, including heart rate and oxygen saturation, are crucial, with adjustments to supplemental oxygen as needed [6]. ICR is an emerging approach that differs from traditional practices, particularly in cord management at birth. Traditional resuscitation often involves immediate cord clamping, which can reduce placental transfusion. In contrast, ICR allows delayed clamping, permitting continued blood flow from the placenta to the newborn during resuscitation. ICR aims to enhance oxygenation and blood volume by utilizing placental blood flow, which can be beneficial in stabilizing the infant's condition before initiating active resuscitation efforts [7]. This method has been associated with improved outcomes, including higher Apgar scores and reduced need for intensive care. While standard practices emphasize rapid intervention, ICR takes a more gradual approach, leveraging the benefits of placental transfusion before transitioning to active resuscitation techniques [8].

Emergence of ICR

ICR is a novel approach in neonatal care that underscores the benefits of delaying cord clamping during the resuscitation of non-vigorous newborns. This technique allows for continued placental transfusion, which may improve outcomes for infants requiring immediate medical intervention. The concept of resuscitating newborns with an intact umbilical cord dates back to ancient times, with references as early as Aristotle. However, it wasn't until the late 20th century that systematic ICR exploration began gaining momentum within the medical community [9]. In the 1990s, delayed cord clamping (DCC) was recognized for its benefits, including improved blood volume and reduced risk of anemia in infants. This recognition laid the foundation for considering ICR as a viable resuscitation option. By the 2000s, research focused on ICR's physiological advantages, particularly when immediate resuscitation was necessary. Studies emphasized that maintaining an intact cord during resuscitation could enhance oxygenation and stabilize cardiovascular function in newborns [9]. Several pivotal studies have shaped the understanding and implementation of ICR. The positive effects of delayed cord clamping on neurodevelopmental outcomes underscored the need for further exploration of ICR as a resuscitation method for non-vigorous infants [10]. Additionally, Katheria et al. (2019) conducted a pilot study assessing the feasibility of ICR in term neonates, finding that less than a third required resuscitation, suggesting the potential for broader application of ICR in clinical settings [11]. Ongoing clinical trials are currently evaluating the safety and efficacy of ICR compared to traditional methods, aiming to address logistical challenges and establish guidelines for routine practice. An interview study explored healthcare professionals' experiences and prerequisites for providing neonatal care during ICR. The emergence of ICR represents a significant shift in neonatal care, informed by historical insights and ongoing research. Innovations in resuscitation tools designed with an intact cord have been developed, fostering multidisciplinary collaboration among healthcare providers to improve neonatal outcomes [12]. As evidence continues to accumulate, ICR may become a standard practice, enhancing the care of non-vigorous newborns and improving their chances of survival and healthy development. The results of ongoing trials and technological advancements will be crucial in overcoming challenges and establishing effective protocols for ICR in clinical settings [13].

Scientific basis of ICR

Physiological Rationale

**Figure 1:**
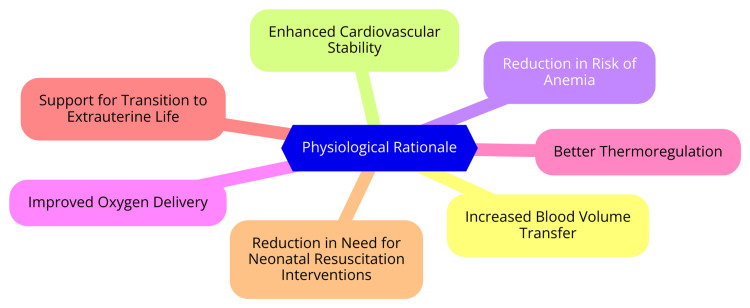
The physiological rationale Image Credit: Dr Sai Bhavani Manchineni

The umbilical cord is a vital link between the fetus and the placenta, enabling the exchange of oxygen, nutrients, and waste products essential for fetal development. Structurally, the cord typically comprises two umbilical arteries and one umbilical vein, all encased in Wharton’s jelly, which provides protection and structural support [14]. The umbilical vein carries oxygenated blood from the placenta to the fetus, while the arteries return deoxygenated blood to the placenta for reoxygenation. This unique arrangement ensures that the fetus receives a continuous supply of oxygen and nutrients while facilitating the removal of metabolic wastes. During fetal life, the umbilical cord plays a critical role in maintaining stable hemodynamics, with blood flow through the cord regulated by various physiological mechanisms. This regulation is crucial for fetal well-being, particularly during the transition to neonatal life at birth [15]. Delaying cord clamping (DCC) has been shown to offer significant physiological benefits for newborns. When the umbilical cord is clamped immediately after birth, the newborn misses out on the essential placental transfusion of blood, which is vital for establishing circulatory stability. Research indicates that delaying clamping allows for a more significant transfer of blood volume from the placenta to the infant, enhancing oxygen delivery and improving hemodynamic stability [16]. This increased blood volume can be especially beneficial for preterm infants, who are at higher risk of complications such as intraventricular hemorrhage (IVH). Studies suggest that DCC can increase a newborn's blood volume by approximately 30-40%, contributing to improved oxygen saturation and overall cardiovascular function immediately after birth [17]. Moreover, delaying cord clamping supports the physiological transition from fetal to neonatal life, a period characterized by significant changes in circulation and respiration. By maintaining placental circulation during this critical phase, DCC helps the newborn establish stable breathing and cardiovascular function without the immediate stress of cord clamping [18]. This practice has been associated with the earlier onset of regular breathing and improved Apgar scores, indicating better overall health at birth. Additionally, DCC has been linked to a reduction in morbidities, including lower rates of anemia, decreased need for blood transfusions, and a reduced incidence of respiratory distress syndrome. The enhanced perfusion and oxygenation DCC provides may also contribute to better infant long-term developmental outcomes [19]. The physiological benefits of DCC are thought to be mediated by several mechanisms, including enhanced lung perfusion and vagus nerve stimulation, which promotes better respiratory function and autonomic regulation in the newborn. The umbilical cord plays a crucial role in neonatal circulation, and delaying cord clamping can significantly improve physiological outcomes for newborns [20]. This practice not only supports the immediate transition to life outside the womb but also has lasting implications for the health and development of infants. As research advances, integrating ICR into clinical practice may further enhance neonatal care [21]. The physiological rationale is illustrated in Figure 1.

Mechanisms and Benefits

ICR represents a groundbreaking approach in neonatal care, particularly for non-vigorous newborns who require immediate resuscitation. By preserving the umbilical cord connection during resuscitation, ICR enables continued placental transfusion, significantly enhancing hemodynamic stability and supporting the newborn's transition to extrauterine life. This method is supported by evidence from animal and human studies, highlighting its various mechanisms and benefits [22]. One of the critical advantages of ICR is its impact on hemodynamics, particularly in terms of blood volume transfer and cardiovascular stability. By allowing continued blood transfer from the placenta to the newborn, ICR increases the infant's blood volume, which is crucial for maintaining cardiovascular stability, especially in infants who may be hypovolemic due to asphyxia or other complications at birth [23]. Studies have shown that delayed cord clamping, as practiced in ICR, facilitates a more stable hemodynamic response, reducing the risk of conditions such as hypoxic-ischemic encephalopathy and persistent pulmonary hypertension of the newborn (PPHN). Furthermore, maintaining an intact umbilical cord during resuscitation has been linked to improved cardiovascular outcomes [24-27]. For instance, research indicates that infants undergoing ICR demonstrate higher heart rates and oxygen saturation levels shortly after birth compared to those who experience early cord clamping. A randomized clinical trial revealed that infants receiving ICR had significantly higher oxygen saturation at 10 minutes post-birth, likely due to enhanced blood flow from the placenta, which helps lower pulmonary vascular resistance and improves perfusion of the lungs and other vital organs [28]. Beyond hemodynamic advantages, ICR also plays a crucial role in thermal regulation and facilitating the transition to extrauterine life. By maintaining the connection to the placenta, ICR helps stabilize the newborn’s temperature during this critical period, reducing the risk of hypothermia when infants are immediately separated from their mothers [29]. The umbilical cord is a conduit for blood and heat, aiding in a smoother transition. Moreover, the physiological changes associated with ICR support the newborn's shift from fetal to neonatal respiratory function. Enhanced blood flow from the placenta assists in lung expansion by distending the alveoli, promoting breathing onset. Studies have found that newborns undergoing ICR tend to initiate breathing earlier than those subjected to early cord clamping, vital for establishing adequate respiration [18]. The benefits of ICR are further corroborated by research in both animal models and human clinical trials. Animal studies, particularly those involving lambs, have shown that delaying cord clamping until after positive pressure ventilation begins leads to improved cardiovascular stability, suggesting that maintaining placental circulation during resuscitation enhances the newborn's physiological adaptation [30]. Human clinical trials have also demonstrated that ICR results in higher oxygen saturation levels, better Apgar scores, and earlier initiation of regular breathing. For example, one study reported that infants resuscitated with an intact cord had significantly better oxygenation and heart rates than those who underwent early cord clamping. Additionally, evidence suggests that ICR may reduce the need for postnatal interventions such as blood transfusions and inotropes by providing adequate blood volume and improving overall stability [30].

Clinical evidence and studies

Key Studies and Trials

ICR has recently gained attention for its potential benefits in neonatal care, particularly for newborns requiring resuscitation. Several studies and clinical trials have investigated the outcomes associated with ICR compared to traditional early cord clamping (ECC) [22]. One significant study is the Nepcord III Trial, a randomized controlled trial conducted at a tertiary hospital in Kathmandu, Nepal, from April to August 2016. This trial focused on late preterm and term infants born vaginally who were non-breathing at birth. The results were compelling, showing that at 10 minutes post-birth, the oxygen saturation (SpO2) levels were significantly higher in the ICR group (90.4%) compared to the ECC group (85.4%) (P < .001). Additionally, the Apgar scores, which assess the newborn's health at 1, 5, and 10 minutes after birth, were higher in the ICR group, indicating better immediate recovery. The study also reported a lower incidence of SpO2 levels below 90% at 10 minutes in the ICR group (44% vs. 100% in the ECC group), highlighting the potential for ICR to improve immediate neonatal outcomes [21]. A systematic review and meta-analysis of various studies further supported these findings, indicating that delaying cord clamping can lead to improved developmental scores in term infants at four years of age and reduced mortality in preterm infants. The physiological benefits observed in these studies included enhanced cardiovascular stability and a reduced risk of complications such as intraventricular hemorrhage, particularly relevant for preterm infants [31]. Feasibility studies have demonstrated that ICR is achievable using different approaches, with research assessing various platforms and equipment designed to maintain intact cord circulation during resuscitation. These studies underscore the practicality of implementing ICR in diverse clinical settings, suggesting that it could be integrated into routine neonatal care with appropriate training and equipment [32]. Regarding outcomes, studies consistently report higher Apgar scores in newborns who undergo ICR than those who experience ECC. The Nepcord III trial found significantly better Apgar scores at all measured time points (1, 5, and 10 minutes) for the ICR group, reinforcing the immediate benefits of this approach. While specific data on anemia incidence related to ICR is limited, the overall improved oxygenation and blood volume transfer associated with ICR suggest potential benefits in reducing the risk of anemia in newborns. Although long-term neurodevelopmental outcomes are still being evaluated, preliminary findings indicate that ICR may positively influence developmental trajectories, particularly in preterm infants [21].

Comparative Analysis

ICR is gaining recognition as a beneficial alternative to traditional early cord clamping in neonatal care. This approach involves delaying the clamping of the umbilical cord during resuscitation efforts, allowing continued placental blood flow to the newborn. Research indicates that ICR can lead to improved initial physiological adaptation, with studies showing higher Apgar scores and better oxygen saturation levels in both term and preterm infants compared to immediate cord clamping, typically performed within the first 30 seconds after birth [12]. Early cord clamping can increase systemic peripheral resistance and arterial pressure, potentially complicating the newborn's transition to life outside the womb. This method has been associated with a higher risk of complications such as intraventricular hemorrhage (IVH) and prolonged asphyxia, particularly in preterm infants [8]. Implementing ICR requires careful coordination and training among healthcare professionals. While the physiological benefits of ICR are well-documented, challenges in its execution include the need for appropriate resuscitation equipment and managing the emotional dynamics of resuscitating a newborn beside the mother. Traditional cord clamping, often simpler to perform in busy clinical settings, may not provide the same advantages for the newborn's health. As healthcare providers become more aware of the benefits of ICR, there is a growing need for training and protocols to facilitate its use in various clinical environments [33]. Regarding clinical outcomes, ICR has demonstrated significant advantages for both term and preterm infants. Studies have shown that ICR can improve early clinical outcomes for term infants without introducing significant adverse effects. By allowing better blood volume transfer from the placenta, ICR enhances the newborn's stability and reduces the likelihood of respiratory distress. The risks associated with ICR in term infants are generally minimal, with challenges primarily logistical rather than physiological [34]. For preterm infants, the benefits of ICR are even more pronounced. Delayed cord clamping has been linked to reduced incidences of IVH and improved blood pressure stability, which are critical factors for this vulnerable population. However, implementing ICR in preterm resuscitation can be complex. The need for immediate intervention in non-vigorous preterm infants creates tension between the urgency of resuscitation and the benefits of delaying cord clamping. Balancing these factors is essential to optimize outcomes for this high-risk group [35].

Current guidelines and recommendations

ICR is gaining recognition in neonatal care, with various international and national health organizations beginning to incorporate its principles into their guidelines. Although the American Academy of Pediatrics (AAP) has not yet established specific guidelines exclusively for ICR, they strongly emphasize the importance of delayed cord clamping in their broader neonatal care recommendations [12]. The AAP advocates for practices that support optimal transition and stabilization of newborns immediately after birth, aligning closely with the objectives of ICR. By delaying cord clamping, healthcare providers can facilitate improved blood volume transfer from the placenta to the newborn, enhancing oxygenation and overall stability [2]. Similarly, the World Health Organization (WHO) has endorsed delayed cord clamping in its guidelines for managing newborns, particularly in low-resource settings. The WHO emphasizes the multiple benefits of this practice, including increased blood volume, improved iron status, and reduced risk of anemia in infants [2]. These recommendations are particularly relevant in contexts where access to healthcare may be limited, as ICR represents a simple yet effective intervention to improve neonatal outcomes. Additionally, various consensus statements from neonatal care experts support ICR as a beneficial practice, underscoring the need for ongoing research to establish standardized protocols. These consensus statements advocate for integrating ICR into routine practice, highlighting its potential to enhance neonatal health outcomes [36]. Despite the growing endorsement of ICR, several practical challenges hinder its widespread implementation in clinical settings. One primary concern is the logistical considerations of maintaining an intact cord during resuscitation [37]. Healthcare facilities may face space constraints in delivery rooms, making it difficult to provide immediate access to resuscitation equipment while ensuring the umbilical cord remains intact. Effective planning and resource allocation are essential to create an environment conducive to ICR, allowing healthcare providers to perform resuscitation efforts without compromising the benefits of delayed cord clamping [37]. Another significant challenge lies in the training and education of healthcare providers. To successfully implement ICR, comprehensive training programs must be developed to equip healthcare professionals with the necessary knowledge and skills. This training should include understanding the physiological benefits of ICR and the specific techniques involved in its execution [38]. Additionally, fostering teamwork among obstetricians, midwives, and neonatal care professionals is crucial to ensuring a coordinated approach to ICR during delivery. Educational programs should prioritize hands-on practice and simulations, allowing staff to gain confidence in performing ICR in real-life scenarios [39]. Current guidelines and recommendations for ICR are outlined in Table 1.

**Table 1:** Current Guidelines and Recommendations for ICR ICR: intact cord resuscitation

Guideline/Organization	Recommendations	Details
American Academy of Pediatrics (AAP) [12]	Delay cord clamping for at least 30-60 seconds after birth.	Emphasizes the benefits of increased blood volume and reduced risk of anemia, particularly in preterm infants.
World Health Organization (WHO) [12]	Encourage delayed cord clamping for 1-3 minutes after birth.	Recommends delaying clamping to enhance iron stores and improve neonatal outcomes, with specific guidance for preterm births.
Royal College of Obstetricians and Gynaecologists (RCOG) [12]	Delay clamping of the umbilical cord for at least 1 minute.	Supports delayed cord clamping to improve neonatal blood volume and reduce the incidence of iron deficiency anemia.
National Institute for Health and Care Excellence (NICE) [12]	Delay umbilical cord clamping for at least 1 minute.	This practice is recommended to support better neonatal outcomes and optimize blood transfer from the placenta.
Canadian Paediatric Society (CPS) [12]	Recommend delayed cord clamping for a minimum of 30-60 seconds, especially in preterm births.	Highlights the importance of delaying clamping to improve outcomes in both term and preterm infants.
Australian and New Zealand College of Obstetricians and Gynaecologists (RANZCOG) [12]	Delay clamping for at least 1 minute and consider longer delays in preterm births.	Encourages delayed cord clamping to improve blood volume and reduce the need for blood transfusions in preterm infants.

Technological and methodological advances

Innovations in Resuscitation Techniques

New technologies and devices supporting ICR: One of the most notable innovations in ICR is the LifeStart Trolley, a mobile bedside resuscitation unit designed to facilitate resuscitation while maintaining an intact umbilical cord. This trolley allows healthcare providers to have essential equipment within proximity during delivery, eliminating the need to move the newborn to a separate resuscitation area. With its adjustable height, integrated heat sources for thermoregulation, and continuous vital sign monitoring capabilities, the LifeStart Trolley has become an invaluable tool in neonatal care [40]. Another significant advancement is the BabySaver, a low-cost mobile resuscitation unit for low-resource settings. The BabySaver provides a stable surface for neonatal resuscitation while ensuring the newborn remains attached to the placental circulation. It includes compartments for storing resuscitation equipment, enabling immediate care at the mother’s bedside, and aiming to reduce neonatal mortality in underserved areas [41]. Purpose-built resuscitation tables are also being developed to address the logistical challenges associated with ICR. These tables are designed to maintain a sterile field while providing easy access to resuscitation tools. Equipped with features that enhance maneuverability and accessibility, they allow healthcare providers to perform necessary interventions without delay, improving the overall efficiency of the resuscitation process [42].

Advances in monitoring and assessment during resuscitation: Alongside these technological advancements, significant progress has been made in monitoring and assessment techniques during resuscitation. Integrating multichannel physiological monitors into resuscitation platforms allows for real-time assessment of vital parameters such as heart rate, oxygen saturation, and respiratory effort. This continuous monitoring is crucial for evaluating resuscitation efforts' effectiveness and making timely interventions [43]. Devices equipped with pulse oximeters and respiratory monitors provide immediate feedback on the newborn's condition, helping healthcare teams assess the need for further interventions. These tools enhance the ability to maintain adequate oxygenation and ventilation during the critical first moments after birth, ensuring that the newborn receives optimal care [44]. Furthermore, innovations such as video cameras for visual monitoring and real-time temperature probes contribute to a more comprehensive approach to neonatal care during resuscitation efforts. These features enable healthcare providers to closely monitor the newborn’s condition, maintain normothermia, and address any complications that may arise during resuscitation [45].

Future directions

The field of ICR is rapidly advancing, with ongoing research focusing on refining techniques and establishing best practices. Recent scoping reviews have highlighted various approaches to ICR, indicating that while many methods are feasible, definitive conclusions on optimal practices are still lacking [46]. A comprehensive review of 2,613 studies, including 18 that explored different equipment and management strategies for ICR, revealed a diverse range of practices across multiple countries. Significant clinical trials are currently underway to evaluate the safety and efficacy of ICR compared to traditional methods such as immediate cord clamping and umbilical cord milking [47]. These trials aim to determine whether ICR can consistently improve outcomes for non-vigorous term and preterm infants who require resuscitation at birth. The potential for ICR to become standard practice will depend on the results of these studies, which are expected to provide critical insights into its clinical benefits [47]. Despite the promising potential of ICR, several challenges and areas for improvement remain. A major obstacle is the logistical and environmental challenges of implementing ICR in hospital settings. Studies have reported difficulties in maintaining a sterile field during resuscitation, particularly when using non-standard equipment. The lack of commercially available sterile tools and the need for effective temperature control present substantial barriers that must be addressed to ensure the safety and efficacy of the practice [48]. Providers have also expressed concerns about accessing necessary resuscitation equipment while performing ICR. Innovations such as purpose-built resuscitation tables are being developed to improve access and ergonomics for healthcare teams during critical moments. Additionally, there is a recognized need for enhanced training for healthcare professionals involved in ICR. Multi-professional team training, including education and debriefing sessions, ensures effective collaboration during resuscitation efforts, enabling teams to work seamlessly under pressure [49]. Another critical area for future investigation is the establishment of standardized guidelines governing ICR practices. The absence of international protocols can lead to inconsistent application across clinical settings [50]. Establishing standardized practices will be crucial for the widespread adoption and effective implementation of ICR. Lastly, while immediate outcomes such as oxygenation and Apgar scores are promising, further research is needed to assess the long-term implications of ICR on neonatal health. Understanding the long-term developmental impacts of ICR remains a critical area for future investigation [49]. Technological and methodological advances in ICR are detailed in Table 2.

**Table 2:** Technological and Methodological Advances in ICR ICR: intact cord resuscitation

Category	Advancement	Description	Impact on ICR
Technological Innovations [46]	Cord Clamping Devices	Advanced devices designed for controlled and precise clamping of the umbilical cord.	Ensures consistency and minimizes the risk of improper clamping.
	Cord Blood Volume Measurement Tools	Tools that accurately measure the volume of blood transferred through the umbilical cord.	Facilitates evaluation of the effectiveness of delayed clamping.
	Neonatal Monitoring Systems	Enhanced monitoring systems that track vital signs and physiological parameters during resuscitation.	Provides real-time data to optimize resuscitation techniques.
Methodological Advances [46]	Enhanced Training Programs	Development of comprehensive training programs for healthcare providers on ICR techniques and benefits.	Improves implementation and consistency in resuscitation practices.
	Standardization of Resuscitation Protocols	Establishment of standardized guidelines and protocols for intact cord resuscitation.	Ensures uniformity and adherence to best practices.
	Simulation-Based Learning	Use of advanced simulation tools for training in ICR and other resuscitation techniques.	Enhances practical skills and readiness of healthcare providers.
	Research on Optimal Timing	Studies focus on the ideal timing for delaying cord clamping to maximize benefits.	Refines protocols and improves patient outcomes.
	Development of Decision Support Tools	Integration of decision support systems to aid clinicians in making informed decisions about ICR.	Supports clinical decision-making and personalized care.

Case studies and clinical experiences

Real-World Applications

ICR has demonstrated its potential benefits in various clinical settings, illustrating its advantages for newborns. A notable case involved a critically ill newborn who was resuscitated with the umbilical cord left intact. This case underscored the physiological benefits of placental transfusion, which can enhance blood volume and oxygenation during the crucial moments after birth. By delaying cord clamping, the healthcare team was able to provide essential support to the infant, showcasing the positive impact of ICR on neonatal outcomes, especially in cases of asphyxia [22]. In addition to individual cases, several pilot studies have examined the feasibility of ICR, particularly for preterm infants. For example, a study involving infants born at 24 to 32 weeks gestation used continuous positive airway pressure during the initial 90 seconds of delayed cord clamping. The findings indicated that ICR could be safely incorporated into neonatal resuscitation protocols without adverse effects, demonstrating its effectiveness in practical settings [51]. Furthermore, feedback from both parents and healthcare providers revealed that, while some clinicians encountered challenges with ICR, a significant majority of parents reported positive experiences during bedside resuscitation. This highlights the benefits for the neonate and the improved parental engagement during critical moments [51]. These experiences emphasize the importance of team training and collaboration in successfully implementing ICR. Effective training programs should enhance communication and teamwork among healthcare professionals, obstetricians, and neonatal teams. Additionally, developing purpose-built resuscitation tables and ensuring easy access to necessary equipment can help address logistical issues, such as maintaining a sterile field and ensuring proper temperature control during resuscitation [52].

Challenges and Controversies

ICR has shown promising benefits in clinical settings, particularly for enhancing neonatal outcomes. A notable case demonstrated the advantages of maintaining an intact umbilical cord during resuscitation. This approach allowed for continued placental transfusion, improving blood volume and oxygenation during the critical moments after birth. By delaying cord clamping, the healthcare team could provide crucial support, highlighting the positive impact of ICR, especially in asphyxia cases [22]. Several pilot studies have further explored ICR's feasibility, particularly for preterm infants. For instance, a study with infants born between 24 and 32 weeks gestation involved continuous positive airway pressure during the initial 90 seconds of delayed cord clamping. The results indicated that ICR could be safely integrated into resuscitation protocols without adverse effects, demonstrating its effectiveness in real-world scenarios [51]. Additionally, feedback from parents and healthcare providers indicated that, despite some challenges clinicians face, most parents had positive experiences with bedside resuscitation, reflecting the benefits of ICR for neonates and parental engagement [51]. These experiences highlight the need for comprehensive team training and collaboration for successful ICR implementation. Effective training programs should enhance communication and teamwork among obstetricians and neonatal teams. Moreover, developing purpose-built resuscitation tables and ensuring ready access to necessary equipment can help overcome logistical challenges, such as maintaining a sterile field and ensuring proper temperature control during resuscitation [52-56].

## Conclusions

In conclusion, ICR represents a transformative approach to neonatal care, offering promising benefits that challenge traditional resuscitation practices. The shift towards delaying cord clamping highlights a growing recognition of the physiological advantages of allowing continued placental blood transfer. This can significantly enhance neonatal outcomes by improving blood volume and reducing the risk of complications such as anemia. The review of current evidence and practices underscores the positive impact of ICR on newborn health, reinforcing its potential to optimize the transition to extrauterine life. However, while the evidence supports the advantages of ICR, continued research and clinical evaluation are essential to address implementation challenges and refine guidelines. By integrating recent advancements and addressing existing gaps, the ongoing evolution of neonatal resuscitation practices can lead to more effective and individualized care for newborns, ultimately contributing to better health outcomes and a higher standard of neonatal care.
